# A study of how immersion and interactivity in MoCap mediate psychological affordances and drive conceptual learning in computer animation courses

**DOI:** 10.3389/fpsyg.2026.1813127

**Published:** 2026-06-09

**Authors:** XinYi Jiang, Zainuddin Ibrahim, Ying Li, MuYing Luo

**Affiliations:** 1 Faculty of Art & Design, Universiti Teknologi MARA, Shah Alam, Malaysia; 2 Faculty of Animation, School of Arts, Anhui Xinhua University, Hefei, China

**Keywords:** computer animation, educational psychology, immersive learning, interaction experience, motion capture

## Abstract

**Introduction:**

Process-level mechanisms remain underexplored in computer animation education. This study reports an initial empirical test and refinement of the Cognitive Affective Model of MoCap Training (CAMMT), which proposes that immersion and action-contingent interactivity shape training through psychological affordances and related cognitive-affective processes.

**Methods:**

Immersion and interactivity were isolated in a 2 × 2 between-subjects experiment (*N* = 117) during a virtual lesson on character motion design. Two-way ANOVAs tested the effects of immersion and interactivity on CAMMT variables and conceptual knowledge gain, and structural equation modeling (SEM) evaluated selected CAMMT pathways.

**Results:**

Action-contingent interactivity robustly increased presence, agency, and multiple training-process variables, and was associated with greater pre-post conceptual knowledge gains. Immersion reliably increased presence and improved training-process experiences but did not yield a consistent advantage for conceptual knowledge gain. Interaction patterns suggested diminishing returns when both features were high, and immersion enhanced agency mainly when MoCap-based interaction was available.

**Discussion:**

The findings provide partial support for CAMMT, clarify how MoCap-based interactivity may shape conceptual learning in a short animation-training lesson, and identify refinements needed for future studies of immersive and interactive technologies in educational psychology and computer animation education.

## 1 Introduction

Performance has long been central to character animation, as Disney veterans observed that “basically, the animator is the actor in the animated films” (Thomas and Johnston, 1995, p. 66). In computer animation courses, students develop these competencies through a continuum of complementary practices, including hand-keyed animation, video reference, rotoscoping, hybrid keyframe/MoCap workflows, and emerging video-input or AI-assisted animation tools. These approaches are not mutually exclusive; in both industry and education, they often coexist as resources for studying, generating, refining, and evaluating motion. Within this broader ecosystem, MoCap turns training into a form of computer puppetry by transforming learners’ movements into character motion in real time, allowing them to “play” animation rather than merely edit it (Shin et al., 2001; Oore et al., 2002). When used as a training medium rather than merely a production shortcut, MoCap can reconfigure learning as an embodied practice in which the learner’s body becomes a primary interface for ideation, performance exploration, and iterative refinement. This creates new opportunities for animation education, but it also raises a more specific research question: how do particular MoCap features shape students’ animation-related learning processes and conceptual understanding?

Researchers have long examined what makes MoCap distinctive and how it can be leveraged in education. For example, Johnson-Glenberg et al. (2014) described a MoCap learning platform as “immersive” and “highly interactive,” using MoCap to track students’ gestures and locomotion as they learn kinesthetically. In this line of work, the educational value of MoCap is not limited to recording movement. Rather, MoCap functions as a delivery mechanism by coupling embodied, multisensory engagement with movement-contingent control, so that learners’ bodies directly shape what happens on the display. MoCap in learning emphasizes two affordances: immersion and interactivity. Jiang et al. (2025) describe these MoCap properties in terms of user-controlled virtual humans, commonly referred to as avatars. Avatars are closely tied to users’ physical selves, fostering a sense of presence and embodiment in virtual environments. By bridging users and the virtual world, avatars support interaction and engagement and enhance perceived authenticity and realism. In turn, these experiences can strengthen learners’ cognitive and creative capabilities. However, in animation education, arguments for MoCap have often been framed as broad media comparisons, such as contrasting MoCap-based workflows with keyframing (Terra and Metoyer, 2007; Wang et al., 2011; Johnson-Glenberg et al., 2014; Mou, 2018). Such comparisons are useful but incomplete because they can imply a dichotomy between tools that are often complementary, and because they typically make immersion, interactivity, practice mode, and feedback vary together. Accordingly, the first objective of this study is to isolate and jointly manipulate immersion and action-contingent interactivity in MoCap training, examining their unique and combined effects on key psychological variables and conceptual animation knowledge gain.

A second limitation reflects an enduring bias toward the “newness” of tools and their technical affordances in animation training, with comparatively limited attention to learners’ cognitive and instructional processes. Such learner neglect in technology-led environments can obstruct learning goal attainment (Chandler, 2009). However, the field currently lacks a mechanism-focused model that explains how MoCap’s core features translate into cognitive-affective training processes in computer animation courses. To address this gap, we adopt the Cognitive Affective Model of Motion Capture Training (CAMMT), proposed by Jiang et al. (2026), as a process-oriented framework that specifies testable pathways linking MoCap features to psychological affordances and downstream training constructs. Because CAMMT was recently proposed and has not yet been widely tested, the present study should be read as an initial empirical test and refinement of the framework rather than as definitive validation.

**Figure 1:**
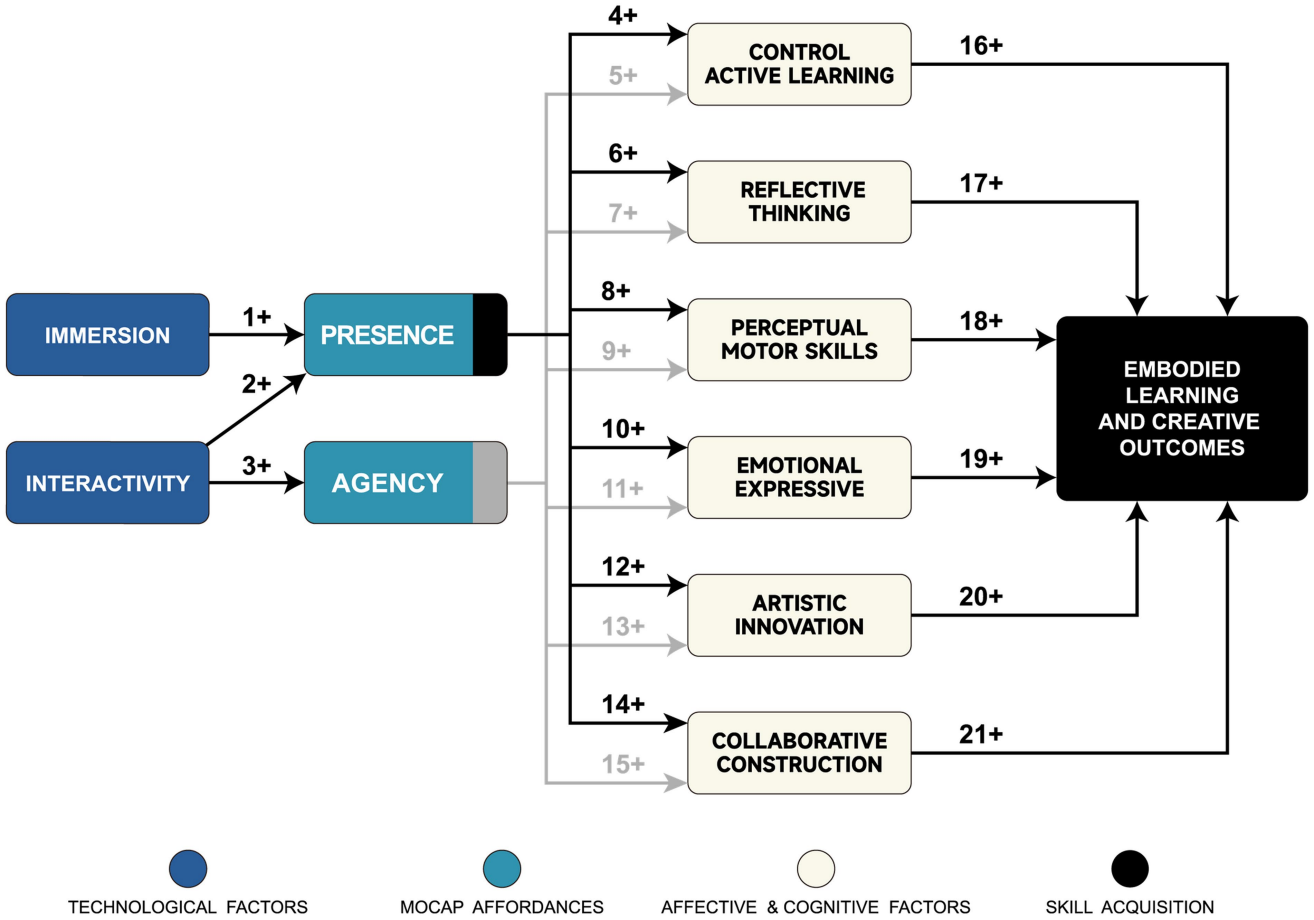
The hypothesized model, based on the CAMMT.

When it comes to training, CAMMT proposes that key advantages and potential constraints of MoCap originate in its two defining features: interactivity and immersion. These features influence training primarily through two psychological affordances: presence, conceptualized here as a performative form of being there in the animation-making situation, and agency, defined as a sense of intentional control and authorship over animated action. Presence and agency are then expected to shape six training-process constructs (depicted in Figure 1 and described in detail below). These constructs, in turn, are proposed to predict MoCap training outcomes. In the present experiment, the measured outcome was conceptual animation knowledge gain; it was not intended to capture the full range of procedural, performative, or creative competence in animation. Because the CAMMT relations are formulated as specific, testable paths, the second objective of this study is to empirically test and refine selected CAMMT pathways using SEM.

In sum, whereas the first research objective addresses the isolated and combined effects of immersion and interactivity, the second objective evaluates their roles within a broader process model of MoCap training in computer animation courses.

### 1.1 Current study

This study describes a media-based experiment built around a virtual lesson on motion principles for character animation, in which immersion and action-contingent interactivity were systematically manipulated. The primary aim was to test the main effects of each feature and their combined effect on the psychological affordances and training-process constructs specified by CAMMT as central to MoCap-based animation training. To isolate these effects, the same core lesson was delivered through four conditions that operationalized a 2 × 2 design: video (low immersion, low interactivity), MoCap (low immersion, high interactivity), VR-video (high immersion, low interactivity), and VR-MoCap (high immersion, high interactivity). Immersion was manipulated using head-mounted displays (HMD) in high-immersion conditions. Interactivity was operationalized as real-time, action-contingent body-to-character control through MoCap-based feedback loops. In the low-interactivity conditions, learners observed the same lesson content and demonstrations without controlling or generating character motion. Because the high-interactivity conditions necessarily involved embodied enactment, the manipulation should be interpreted as action-contingent MoCap interactivity as implemented through practice, not as a complete separation of technological interactivity from all forms of active learning.

A further aim was to empirically evaluate and refine the CAMMT pathway model for training in immersive, performance-oriented contexts. Specifically, the analysis examined whether immersion and interactivity primarily affect training through presence and agency, which then predict subsequent training-process constructs and conceptual knowledge gain. These relationships were assessed using structural equation modeling (SEM) based on the measured variables. This objective was motivated by a desire to empirically chart the training process in technology-driven animation learning environments, shifting attention from evaluating only final animation products to explaining mechanisms that unfold during training. The design therefore provides evidence relevant to instructional design while recognizing that classroom animation assessment typically also requires practical exercises, artifacts, critique, and expert evaluation. In the following, we describe the background to the study along with our hypotheses and predictions.

## 2 Background

The CAMMT is a research-based theoretical model that describes how learning and skill development unfold during MoCap-based training in computer animation courses, with an explicit focus on the training process rather than only final animation products. The model was developed in response to scholars’ calls for stronger theory to guide MoCap training research and development (Wang, 2022; Reuter and Schindler, 2023; Jiang et al., 2025), given that existing studies often emphasize finished outputs and rely on broad media comparisons (e.g., Mou, 2018) that conflate multiple technological, pedagogical, and practice-related affordances. In animation education, these comparisons should be situated within a broader landscape that also includes video reference, rotoscoping, hybrid keyframe/MoCap workflows, and newer video-input or AI-assisted systems. In the following sections, we outline CAMMT’s assumptions and organize prior work relevant to its components, and we use this foundation to derive testable predictions for the present feature-based experiment (see Section 2.6).

CAMMT builds on a body of technology-enhanced learning research that conceptualizes media effects as largely indirect: media features influence learning outcomes by first shaping intermediate psychological states and processes, such as presence, motivation, perceived control, and perceived usability (Lee et al., 2010; Johnson-Glenberg et al., 2014; Makransky and Petersen, 2019). In immersive-learning research, this process-oriented perspective is commonly operationalized using mediation analyses and SEM, which has shown that core technological characteristics, including immersion and interactivity, often affect learning through affective and cognitive pathways rather than through direct effects on performance measures (Petersen et al., 2022). Extending this logic to MoCap-based animation training, CAMMT assumes that MoCap does not inherently “cause” learning. Instead, its affordance influences what learners do and experience during practice. When aligned with instructional goals, MoCap can amplify engagement, authorship, reflection, and embodied exploration; when poorly aligned, the same affordances may introduce friction or distraction that constrains these processes and, ultimately, training outcomes.

Theoretically, CAMMT can be situated within a broader family of frameworks that integrate cognitive and affective processes in learning from interactive media. This includes the Cognitive-Affective Theory of Learning with Media (Moreno and Mayer, 2007) and immersive-learning process models such as CAMIL (Petersen et al., 2022), which converge in treating attention allocation, cognitive load, and learners’ motivational and emotional responses as central determinants of learning outcomes. CAMMT also draws on embodied cognition accounts, which argue that knowledge and understanding are grounded in sensorimotor systems and situated action (Wilson, 2002; Barsalou, 2008), as well as design and creative cognition perspectives that portray learning as emerging through iterative cycles of externalizing, inspecting, and revising ideas through artifacts and prototypes (Schon and Wiggins, 1992; Dow et al., 2010). In animation training, these assumptions align closely with studio-based pedagogies in which competence develops through repeated cycles of making, critique, and revision. This orientation is particularly compatible with MoCap, where motion is produced through bodily performance and progressively refined through repeated enactment and feedback.

### 2.1 CAMMT’S underlying conception of computer animation training

CAMMT embraces a constructivist and design-oriented perspective because computer animation training is transdisciplinary and practice-based. Students must integrate motion principles, tool-mediated production, and expressive performance decisions within the same task, and this integration cannot be achieved through passive reception of rules alone. Instead, learners construct competence through iterative cycles of making, feedback, and revision, which makes a process-oriented account of training necessary. Within this stance, MoCap is not assumed to improve learning by itself or to replace other animation practices. Rather, it shapes training by influencing what learners experience and do during practice. This provides the principle for construct selection: CAMMT prioritizes variables that are proximal to the medium and central to training, namely presence and agency as psychological affordances, and downstream mechanisms that capture how learners engage, reflect, calibrate embodied skills, and develop expressive and creative performance in studio-like practice. Causally, the model specifies a mediated pathway from MoCap features (immersion and interactivity) to presence and agency, from these affordances to training-process mechanisms, and from those mechanisms to learning and creativity outcomes. The scope of CAMMT is bounded to MoCap-supported instruction in computer animation courses where learners enact motion and receive feedback; it is not intended as a general claim of media superiority, nor as a model that primarily explains production quality through representational fidelity or other non-instructional pipeline factors.

As Figure 1 illustrates, the variables included in CAMMT can be organized into several linked sections. From left to right, the model begins with MoCap features, proceeds to the core psychological affordances, and then to downstream training process constructs and outcome variables. In the following sections, we review each part of the model in this order. Figure 1 also summarizes the hypothesized relations among the variables. Each path is numbered to match the corresponding prediction and is shown with its expected directional effect, which is outlined in the following text.

### 2.2 MoCap features

The leftmost section of CAMMT contains the MoCap features examined in this study, namely immersion and interactivity (both defined above). Interactivity is treated here as a control factor, and representational fidelity is held constant to limit the number of experimental conditions. Importantly, CAMMT distinguishes these focal features from pipeline-related characteristics, such as motion data accuracy, mapping precision, and character setup; in the present study, these fidelity-related aspects are controlled as background conditions rather than modeled variables, enabling a cleaner test of the unique and combined effects of immersion and interactivity.

### 2.3 Presence and agency

The next part of the model introduces presence and agency as two psychological affordances through which immersion and interactivity are translated into trainees’ subjective experience during MoCap practice. Presence is commonly defined as the experience of “being there” in a mediated environment, and it is not inherently tied to any single technology but is experienced by the user (IJsselsteijn and Riva, 2003). CAMMT frames presence in MoCap training as performative presence: across cycles of movement enactment and observation–revision, learners feel situated inside the action situation and form an immediate perception with character motion through the body (Ratan, 2013).

Agency refers to the subjective experience of initiating and controlling one’s actions and, through them, effects in the world, emphasizing authorship and intentional control rather than mere usability or operational proficiency (Moore and Fletcher, 2012). In interactive environments, agency is strengthened when action control is responsive and consequential, and when sensorimotor predictions align closely with perceived feedback (Johnson-Glenberg, 2018). In MoCap training, such alignment is supported by near real-time character feedback that closes the loop between movement intention, bodily execution, and animated consequences, making learners more likely to treat adjustments as design decisions and enabling exploration and creative variation. Hence, this subsection predicts: 1: Immersion will positively predict presence, 2: Interactivity will positively predict presence and 3: Interactivity will positively predict agency.

### 2.4 Six affective and cognitive variables

CAMMT argues that the value of MoCap should not be evaluated solely by final animation products but should be explained through what happens during the training process. Accordingly, the model connects presence and agency to six training-process constructs that are highly relevant to character animation training: control active learning, reflective thinking, perceptual–motor skills, emotional expressiveness, artistic innovation, and collaborative construction.

Control active learning describes the extent to which learners engage in goal-directed exploration, experimentation, and adjustment during practice (Lee et al., 2010). In CAMMT, control is not only “I can operate the system,” but also “I can lead the generation and revision of motion solutions.” When presence is stronger, learners are more likely to sustain attentional involvement in the training situation and remain engaged in continued exploration (Witmer and Singer, 1998). When agency is stronger, learners are more likely to experience motion adjustment as intentional, self-authored action and thus interpret revisions as their own creative decisions (Moore and Fletcher, 2012). Hence, this subsection predicts 4: Presence will positively predict control active learning. 5: Agency will positively predict control active learning.

Reflective thinking in MoCap-based animation training enacted through short perform–view–revise cycles, where bodily performance externalizes motion ideas, replay enables evaluation, and revisions refine intent, consistent with design cognition accounts of iterative externalization and refinement (Cross, 2011). Stronger presence should make motion cues more salient and thus more likely to trigger reflection (Witmer and Singer, 1998). Stronger agency will increase learners’ willingness to translate reflection into iterative revision and re-attempts (Moore and Fletcher, 2012; Code, 2020). Therefore, this subsection predicts 6: Presence will positively predict reflective thinking. 7: Agency will positively predict reflective thinking.

Perceptual motor skills refer to increasingly precise control of motion timing and dynamics (e.g., rhythm, weight, force, and trajectory) by integrating sensory feedback with bodily control (Shadmehr et al., 2010). Stronger presence should heighten attentional involvement in the action situation, making spatiotemporal cues more salient for calibration, whereas stronger agency, as a heightened sense of control over action outcomes, is associated with greater learnability in sensorimotor adaptation and thus more efficient feedback-based refinement (Wen et al., 2021). Therefore, this subsection predicts 8: Presence will positively predict perceptual motor skills. 9: Agency will positively predict perceptual motor skills.

Emotional expressive refers to learners’ ability to convey affective states (e.g., joy, tension, fear) through bodily movement and character performance. Design research suggests that bodily movement can be a powerful source of inspiration for developing emotionally expressive systems (Ståhl, 2005). Similarly, Kiiski et al. (2015) argue that in the context of animation, emotional expressiveness plays a crucial role in transforming motion into meaning, enabling learners to imbue characters with personality and emotional nuance. Consistent with embodied perspectives that link affect and cognition to sensorimotor experience, MoCap makes expressive intent observable and adjustable, supporting the capture and refinement of affective movement qualities (Crane and Gross, 2007; Ennis et al., 2013; Bennett and Kruse, 2015). Therefore, this subsection predicts 10: Presence will positively predict emotional expression. 11: Agency will positively predict emotional expression.

Artistic innovation describes learners’ capacity to generate and refine novel yet appropriate motion ideas, producing movement solutions that are original while still fitting character, narrative, and stylistic constraints (Wang and Zhong, 2024). Presence immerses learners in a performative context that supports spontaneity and full-body engagement. When presence is high, learners treat the task as lived performance rather than detached manipulation, which encourages risk-taking, improvisation, and alternative interpretations. Evidence indicates that feeling inside the animation space increases the likelihood of creative risk-taking and expressive variation (Mou, 2016). Agency with intentional control and immediate visual feedback, learners can shape movement with clear expressive intent and iterate rapidly. This freedom encourages departures from standard movement templates toward original performance styles (Chan et al., 2010). Therefore, this subsection predicts 12: Presence will positively predict artistic innovation. 13: Agency will positively predict artistic innovation.

In the present individual experiment, collaborative construction is best understood as perceived collaborative construction potential rather than actual collaboration during the session. The construct refers to learners’ perception that a training approach could support sharing, discussion, co-construction, and knowledge exchange in animation-learning contexts (Fischer et al., 2002). This clarification is important because participants were instructed not to interact with one another during the experiment. There is growing evidence that technology-based training environments can be especially effective when paired with face-to-face interactions that enhance collaboration and knowledge sharing (Park et al., 2019; Cila, 2022). Immersive VR/AR collaboration enables shared experiences and offers high realism, interactivity, and flexibility across domain-specific spaces, and learning gains may reflect social cohesion and cognitive developmentalist processes (Johnson-Glenberg et al., 2014; Devigne et al., 2017; Chheang et al., 2021). Manaf and Arshad (2020), in their study on a team-learning MoCap project for rigging operations, suggested that in multi-user or team-based MoCap settings, embodied presence enhances the interpersonal dimension of performance, allowing learners to synchronize actions and co-create expressive scenes together. Hence, this subsection predicts 14: Presence will positively predict perceived collaborative construction potential. 15: Agency will positively predict perceived collaborative construction potential.

### 2.5 Embodied learning and creative outcomes

The rightmost part of the model represents training outcomes. CAMMT broadly distinguishes between (1) animation knowledge learning (i.e., understanding principles of character motion and motion-design rules, and the ability to transfer and apply them), and (2) motion design creativity (reflected in final products or artifacts). The present study measured the first component through conceptual knowledge gain and did not directly assess final motion-artifact quality. Two recently published systematic reviews found that MoCap has greater potential for learning than traditional media approaches (Reuter and Schindler, 2023; Jiang et al., 2025). Furthermore, evidence suggests that specially designed immersive MoCap systems are particularly suitable for training grounded in motion perception and performance, such as dance, sports and music (Chan et al., 2010; Liu et al., 2020; Campo et al., 2023).

CAMMT further assumes that the six training-process constructs do not merely describe whether the experience “feels good,” but also predict training outcomes. Accordingly, this subsection proposes the following predictions: 16: Control and active learning will positively predict conceptual knowledge gain. 17: Reflective thinking will positively predict conceptual knowledge gain. 18: Perceptual-motor skills will positively predict conceptual knowledge gain. 19: Emotional expressiveness will positively predict conceptual knowledge gain. 20: Artistic innovation will positively predict conceptual knowledge gain. 21: Perceived collaborative construction potential will positively predict conceptual knowledge gain.

### 2.6 Hypotheses on immersion and interactivity

As mentioned previously, many prior studies of animation training and embodied media have relied on broad media comparisons (e.g., MoCap vs. keyframing) that confound multiple technological and pedagogical factors. This study’s design makes it possible to isolate and examine the unique and combined effects of immersion and action-contingent interactivity on CAMMT-relevant variables. Based on the research reviewed above and the general assumption that higher immersion and higher interactivity tend to strengthen learners’ experience of embodied engagement, authorship, and process-relevant training mechanisms, it is predicted that immersion and interactivity will have positive effects on all outcome and process variables investigated in this study. Consequently, a total of nine hypotheses, split into a (signifying immersion) and b (signifying interactivity), are proposed in Table 1.

**Table 1:** Overview of proposed hypotheses (1–9).

Hypothesis	Predicted effect
Hypothesis 1	There will be a positive effect of immersion(1a)and interactivity(1b)on Agency
Hypothesis 2	There will be a positive effect of immersion(2a)and interactivity(2b)on Presence
Hypothesis 3	There will be a positive effect of immersion(3a)and interactivity(3b)on Control Active Learning
Hypothesis 4	There will be a positive effect of immersion(4a)and interactivity(4b)on Reflective Thinking
Hypothesis 5	There will be a positive effect of immersion(5a)and interactivity(5b)on Perceptual Motor Skills
Hypothesis 6	There will be a positive effect of immersion(6a)and interactivity(6b)on Emotional Expressive
Hypothesis 7	There will be a positive effect of immersion(7a)and interactivity(7b)on Artistic Innovation
Hypothesis 8	There will be a positive effect of immersion(8a)and interactivity(8b)on Collaborative Construction
Hypothesis 9	There will be a positive effect of immersion (9a) and interactivity (9b) on conceptual knowledge gain

## 3 Materials and methods

### 3.1 Sample

A total of 117 undergraduate animation students from a comprehensive university in China participated in the experiment as part of a mandatory course in 3D character animation design. The author affiliations listed above reflect the authors’ institutional affiliations and should not be interpreted as the data-collection site. The vast majority of participants were between 19 and 22 years old. Because the sample did not include non-native speakers, the experimental introduction and learning materials were presented in the participants’ native language. All participants had received prior training in Autodesk Maya, ensuring a shared baseline of 3D animation software experience across experimental conditions. Participants in the two VR conditions were relatively inexperienced VR users: most had used VR zero times before (77), some had used it once (22), and some had used it two times or more (18); no participant reported using VR more than 5 times prior to the study.

### 3.2 Procedures

**Figure 2:**
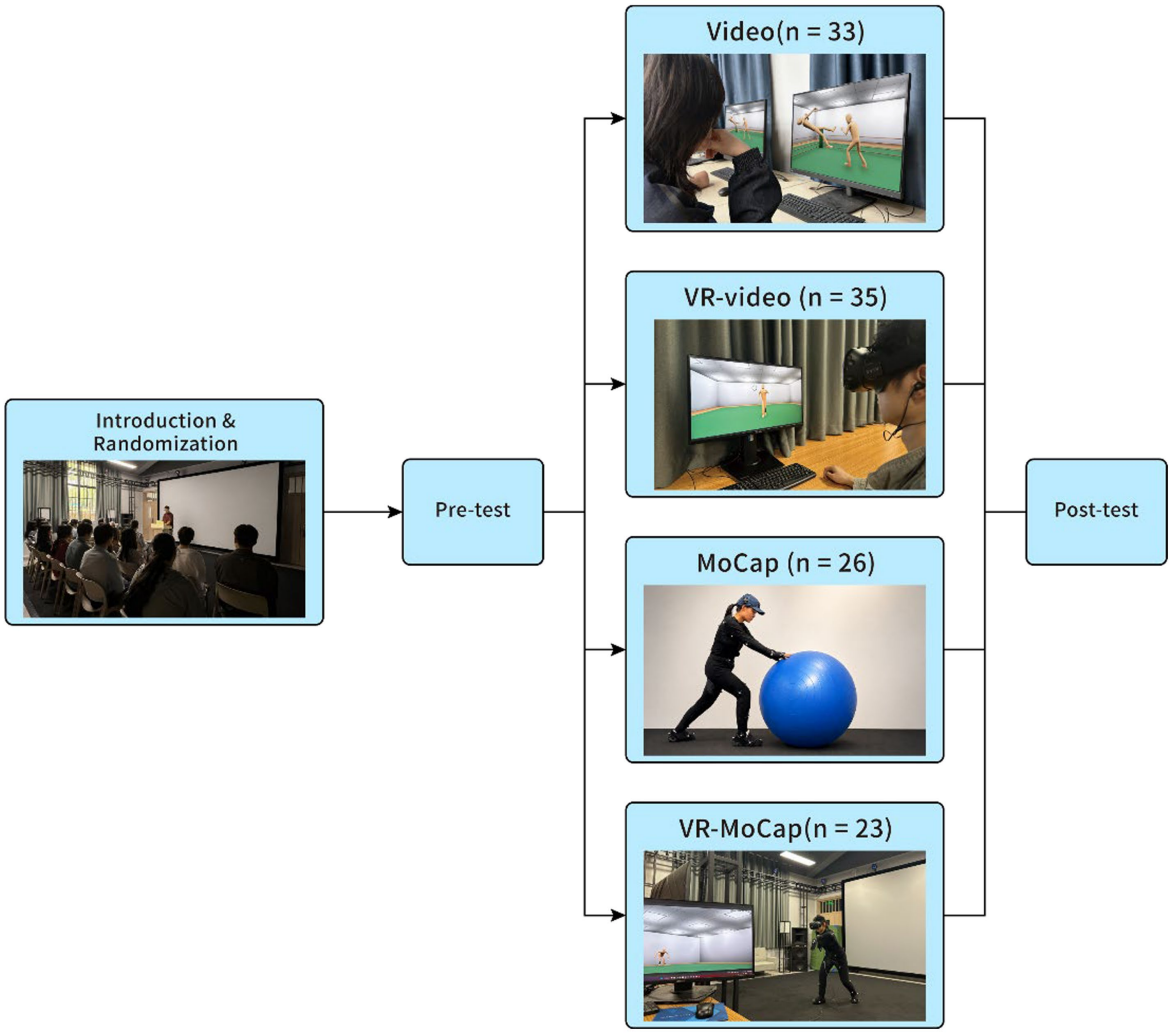
Overview of training procedures.

An overview of the study procedure is given in Figure 2. Participants were drawn from four seminar sections within a compulsory undergraduate program in 3D character animation, with each section comprising approximately 20 to 30 students. The same procedure was applied across all groups. Before random assignment, participants reviewed and signed an informed-consent form that provided a general description of the study and explained their rights as research participants. They were then randomly assigned to one of four conditions: VR-MoCap (*n* = 23), VR-video (*n* = 35), MoCap (*n* = 26), or video (*n* = 33). These four conditions implemented the experimental manipulation of immersion and interactivity (see Table 2). Following allocation, each participant received a unique ID and completed a pre-test measuring prior conceptual knowledge of character motion principles and collecting demographic information, including age, gender, prior experience, and relevant training background.

**Table 2:** Levels of immersion and action-contingent interactivity by condition.

Condition	Immersion	Action-contingent interactivity
Video	Low	Low
VR-video	High	Low
MoCap	Low	High
VR-MoCap	High	High

At the designated location, participants completed a virtual training lesson on character animation motion design, which served as the primary learning material. Participants in each condition engaged with the lesson via their respective medium. In the high-interactivity conditions (MoCap and VR-MoCap), learners actively generated character motion through MoCap-based action-feedback interaction during training. In the low-interactivity conditions (video and VR-video), learners watched a pre-recorded lesson demonstrating the same content and an optimal run-through of the training material, without the ability to control or generate motion. Thus, the high-interactivity manipulation included embodied enactment and immediate system feedback, whereas the low-interactivity manipulation provided observational exposure to the same conceptual content. After the lesson, participants completed the post-test assessing conceptual knowledge again and collecting subjective measures of key CAMMT constructs. Participants were instructed not to take notes and not to interact with one another during the experiment.

To make the condition features explicit, all four groups received the same conceptual lesson content and demonstration sequence. The video group viewed the lesson on a monitor without action control; the VR-video group viewed the same content through an HMD without action control; the MoCap group generated character motion through real-time body-to-character mapping while viewing the output on a large display; and the VR-MoCap group combined HMD immersion with real-time MoCap control. Thus, the interactivity manipulation captured learner movement enactment, action-contingent feedback, and the opportunity to revise motion through performance.

### 3.3 Materials

#### 3.3.1 Virtual training material

**Figure 3:**
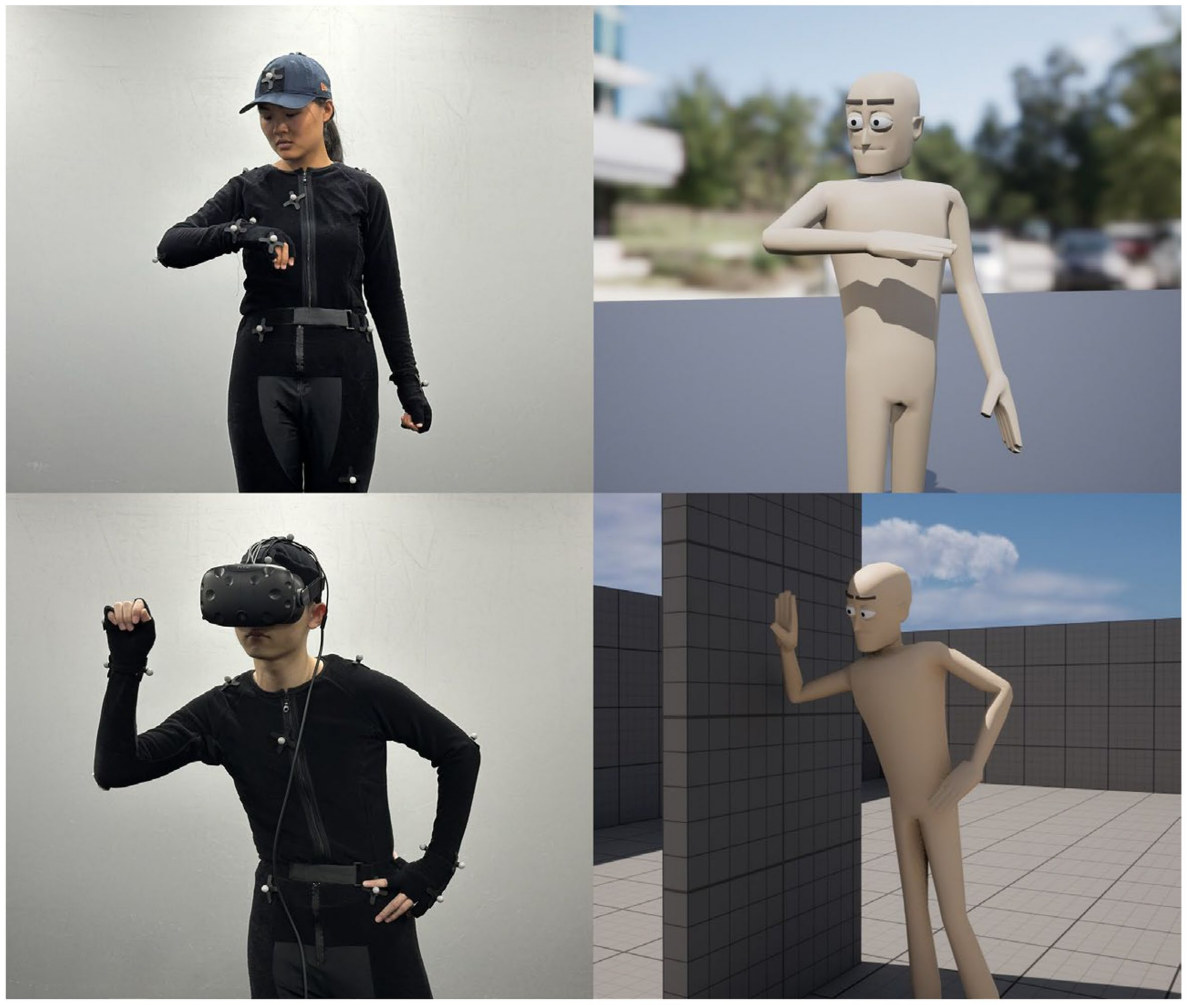
MoCap system setup and calibration.

The core training material consisted of a virtual lesson on principles of character animation and motion design, developed in Unreal Engine 5.4. All digital assets used in the lesson (e.g., character animations, models, and environmental elements) were sourced from free assets available on the Unreal Marketplace and other platforms to support reproducibility and appropriate asset licensing for research use. The lesson was designed around Disney animation principles and motion design knowledge, with the level of difficulty targeted to undergraduate animation students at the corresponding year level. The instructional sequence started with an introduction to core animation principles and then moved to practical guidance on motion design, such as how to design distinctive walk styles (e.g., swaggering vs. sneaking) and how to enhance the appeal and expressiveness of character actions through timing, weight shifts, and pose organization. Four versions of the same virtual lesson were created to operationalize the experimental manipulation of immersion and action-contingent interactivity while holding the conceptual content as constant as possible (Figure 3).

Regarding duration, the core instructional content lasted approximately 10 min in all four conditions. Total session time differed because the MoCap and VR-MoCap conditions required additional equipment donning and calibration. The video and VR-video sessions took approximately 10 min in total, the MoCap condition took approximately 15 min on average, and the VR-MoCap condition took approximately 20 min because it required setup and calibration of both systems; trained volunteers assisted with setup in the VR-MoCap condition. These differences primarily reflected equipment preparation rather than additional instructional content. However, because setup time and active engagement time were not logged as fully separate variables, time-on-task and novelty effects should be considered when interpreting the results.

#### 3.3.2 Devices

Four presentation formats, each representing a distinct combination of action-contingent interactivity and immersion, were used to deliver the virtual learning material (see Table 2). All MoCap and VR equipment was based on an OptiTrack optical tracking system. The experiment was conducted in a lab space of approximately 8 m × 7 m, equipped with 12 OptiTrack PrimeX 22 cameras. The high-immersion conditions (VR-video and VR-MoCap) employed an HTC VIVE head-mounted display (HMD). In the VR-video (high immersion/low interactivity) condition, participants wore the HMD only, whereas in the VR-MoCap (high immersion/high interactivity) condition, participants wore both the motion capture suit and the HMD. In the MoCap (low immersion/high interactivity) condition, participants wore the motion capture suit only and viewed the instructional content on a 60-inch TV. Finally, the video (low immersion/low interactivity) condition used a 22-inch 2 K monitor. Participants used in-ear headphones in the VR-MoCap, VR-video, and video conditions.

#### 3.3.3 Pre-test

**Figure 4:**
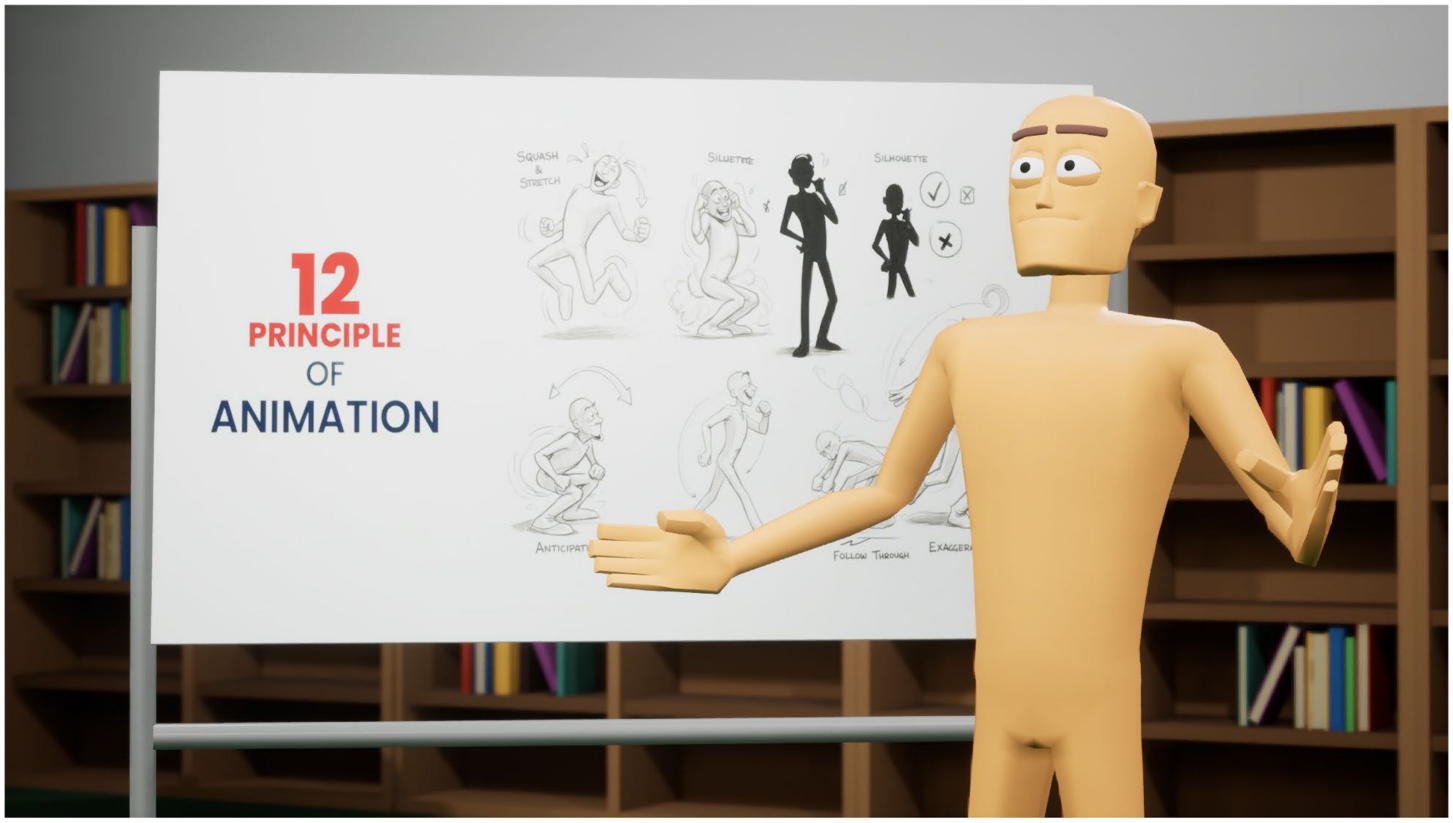
Screenshot of the virtual training scene.

The pre-test collected participants’ demographic information and assessed baseline levels of prior animation software experience and animation knowledge. The experience questionnaire measured self-reported proficiency with animation-related software using a five-point Likert scale ranging from very unskilled to very proficient. The knowledge questionnaire consisted of multiple-choice items targeting core concepts in Disney animation principles and character motion design. Example items included “What does anticipation help achieve in character animation?” and “What is the purpose of exaggeration in character animation?” Pre-test knowledge scores were calculated as the number of correct responses and were re-assessed at post-test using the same or equivalent items to evaluate improvement in conceptual knowledge learning (Figure 4).

#### 3.3.4 Post-test

The post-test assessed participants’ post-training conceptual animation knowledge and measured two CAMMT-based psychological affordances, presence and agency, along with six cognitive-affective constructs. The agency scale was adapted from Polito et al. (2013); e.g., “During the training, my experiences and actions were under my control”. The presence scale was adapted from Makransky et al. (2017); e.g., “The virtual environment seemed real to me”. The control and active learning scale was adapted from Lee et al. (2010); e.g., “Based on the real-time feedback provided by this training approach, I actively adjust my movements to improve animation outcomes”. Reflective thinking was assessed using items adapted from Maor and Fraser (2005); e.g., “During the training, I frequently reflect on how my movements influence the resulting animation”. The perceptual-motor skills scale was adapted from Rovai et al. (2009); e.g., “The training system enhances my spatial perception abilities in animation creation”. The emotional expressiveness scale was adapted from Kring et al. (1994); e.g., “Engaging in animated performance during the training makes it easier for me to form an emotional connection with the character”. The artistic innovation scale was adapted from Cherry and Latulipe (2014); e.g., “This training approach opens up new possibilities for character animation design”. Because participants did not collaborate during the experiment, the collaborative construction scale was interpreted as perceived collaborative construction potential and was adapted from So and Brush (2008); e.g., “Sharing this training approach with others helps me better understand character movement.”

#### 3.3.5 Statistical analyses

For this study, all statistical analyses were conducted using SPSS in conjunction with AMOS. Specifically, the two-way ANOVA was performed in SPSS (v.26), while SEM was implemented using AMOS. In this study, *p*-values < 0.05 were considered statistically significant.

## 4 Results

### 4.1 Quality and measures

For all CAMMT-based measures (i.e., all questionnaire scales except conceptual knowledge learning), Cronbach’s alpha was used as an indicator of scale quality. As recommended by DeVellis and Thorpe (2021), *α* levels between 0.80 and 0.90 were considered indicative of very good internal consistency. The presence scale had a Cronbach’s α of 0.88. The agency scale had a Cronbach’s α of 0.93. The control active learning scale had a Cronbach’s α of 0.84. The reflective thinking scale had a Cronbach’s α of 0.88. The perceptual-motor skills scale had a Cronbach’s α of 0.92. The emotional expressiveness scale had a Cronbach’s α of 0.81. The artistic innovation scale had a Cronbach’s α of 0.88. The perceived collaborative construction potential scale had a Cronbach’s α of 0.89. Item-level reliability diagnostics (e.g., α-if-item-deleted) were inspected for each scale; however, no item deletion was judged necessary to improve psychometric quality without reducing theoretical coverage. Because item CC_5 referred to actual collaboration despite the individual protocol, this item should be interpreted cautiously and checked in future sensitivity analyses. Accordingly, all items were retained in the final scoring.

The knowledge test was edumetric, in that it was intended to capture conceptual learning as the change in performance from pre-test to post-test. Accordingly, it was evaluated using the edumetric criterion of the mean pre-to-post gain (Carver, 1974). The results indicated an average gain of 8.76 points (SD = 11.18) on the 1–100 scale (Pre-test M = 79.62, Post-test M = 88.38), with gains ranging from −20 to 40, suggesting that the test was sensitive to both improvement and individual variability in conceptual knowledge learning.

### 4.2 Group differences at pre-test

Therefore, we conclude that the assigned groups were not fully equivalent with respect to demographic characteristics, and the gender imbalance should be considered when interpreting group comparisons.

### 4.3 Two-way ANOVAs of interactivity and immersion

**Figure 5:**
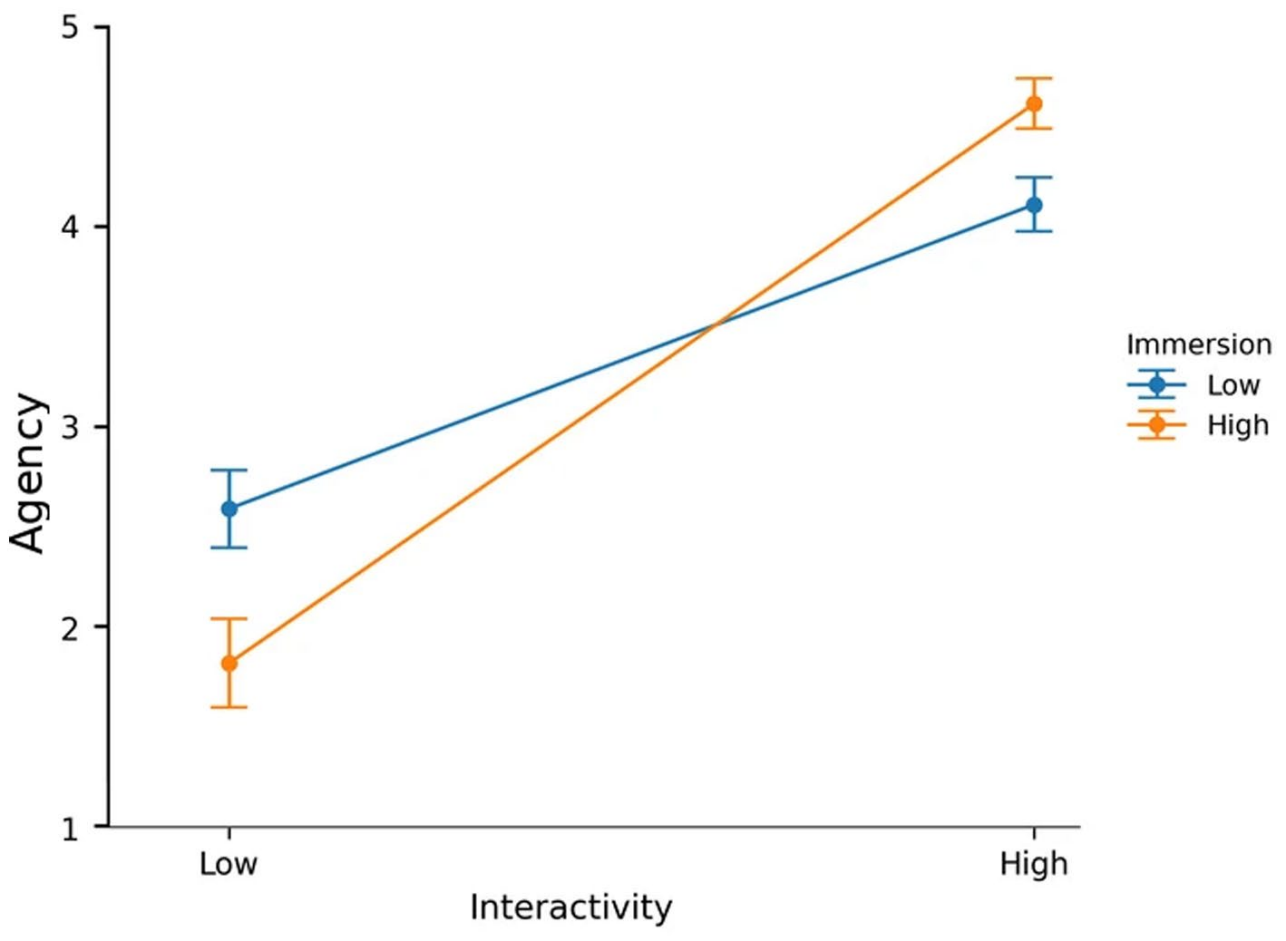
Agency as a function of interactivity and immersion (95% confidence intervals).

**Figure 6:**
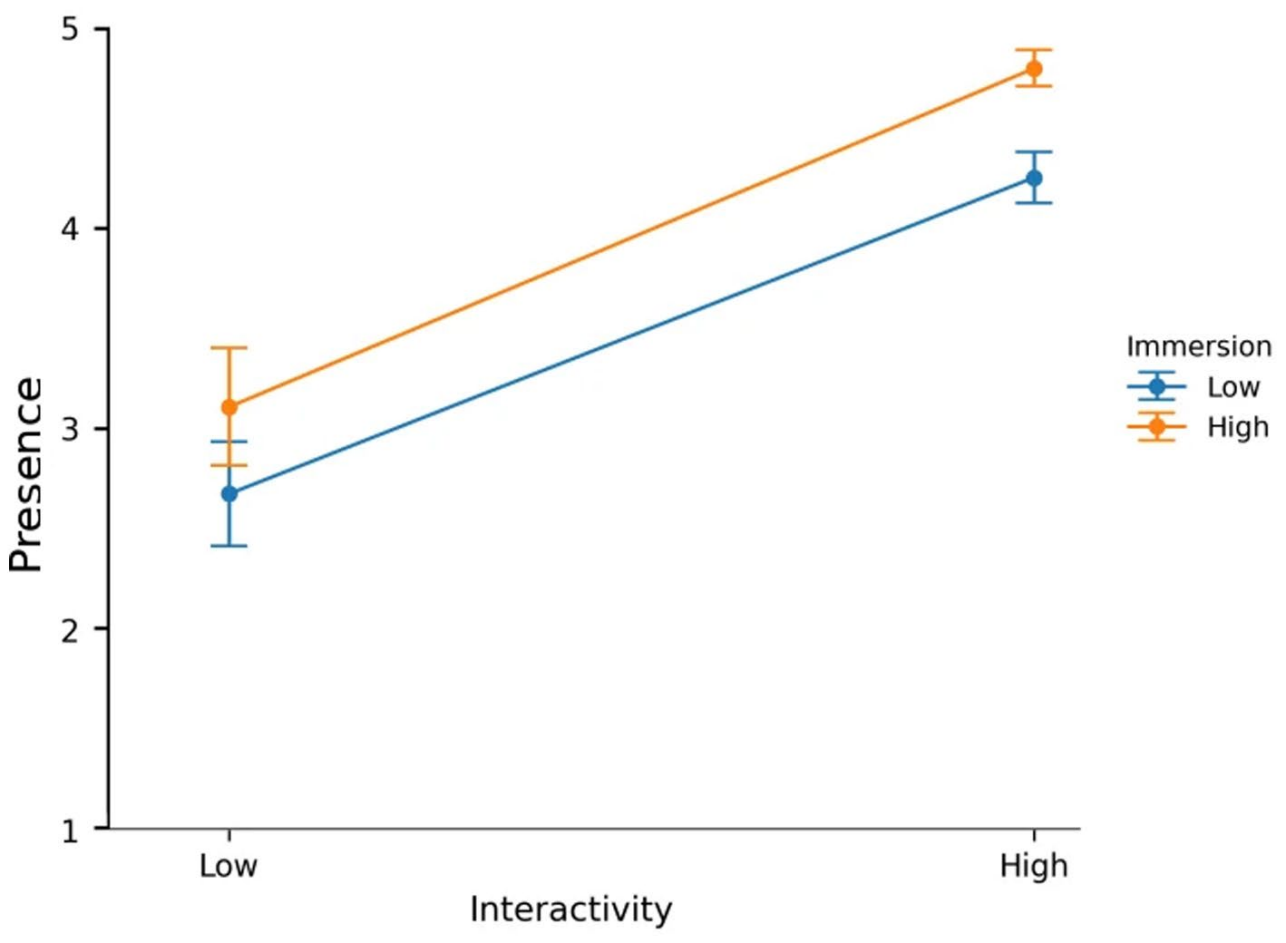
Presence as a function of interactivity and immersion (95% confidence intervals).

Because CAMMT identifies presence and agency as the key psychological affordances, only their interaction plots are shown in Figures 5, 6.

As shown in Figure 5, there was a statistically significant interaction between the effects of immersion and interactivity on agency, *F*(1, 113) = 45.91, *p* < 0.001, ηp^2^ = 0.29, with a larger interactivity effect under high than low immersion (d = 5.21 vs. 3.27). Both main effects were significant (*F*(1, 113) = 517.91 and 6.57; *p* < 0.001 and 0.012), but immersion was not uniformly positive: it reduced agency under low interactivity (VR-video < Video; d = −1.28) yet increased agency under high interactivity (VR-MoCap > MoCap; d = 1.60). Thus, H1b was supported, whereas H1a was supported only conditionally when MoCap interaction was present.

As shown in Figure 6, there was no statistically significant interaction between immersion and interactivity on presence, *F*(1, 113) = 0.22, *p* = 0.643, ηp^2^ = 0.002, indicating that the two factors contributed to presence in an approximately additive manner. In contrast, the main effects of interactivity and immersion on presence were both significant, *F*(1, 113) = [187.78; 16.67], *p* < 0.001, ηp^2^ = [0.62; 0.13], respectively. Consistent with the absence of interaction, the effect of interactivity on presence was similarly large under both immersion levels (low immersion: d = 2.67; high immersion: d = 2.49). Thus, the hypotheses that immersion and interactivity would have positive effects on presence were confirmed (Table 3).

**Table 3:** Overview of the results.

Variable	Low im.		High im.		Main effect im.	Main effect int.	Inter-action
	Low int.	High int.	Low int.	High int.			
Agency	2.59 (0.54)	4.11 (0.33)	1.82 (0.65)	4.61 (0.29)	*p* = 0.012	***p* < 0.001**	***p* < 0.001**
Presence	2.67 (0.74)	4.25 (0.32)	3.11 (0.85)	4.79 (0.21)	***p* < 0.001**	***p* < 0.001**	*p* = 0.643
Control active learning	3.17 (0.50)	4.35 (0.38)	4.37 (0.45)	4.70 (0.20)	***p* < 0.001**	***p* < 0.001**	***p* < 0.001**
Reflective thinking	2.99 (0.73)	4.32 (0.37)	4.27 (0.48)	4.64 (0.21)	***p* < 0.001**	***p* < 0.001**	***p* < 0.001**
Perceptual motor skills	2.85 (0.79)	4.26 (0.31)	4.26 (0.50)	4.70 (0.26)	***p* < 0.001**	***p* < 0.001**	***p* < 0.001**
Emotional expressiveness	3.43 (0.52)	4.28 (0.32)	3.88 (0.52)	4.70 (0.26)	***p* < 0.001**	***p* < 0.001**	*p* = 0.893
Artistic innovation	3.07 (0.50)	4.38 (0.33)	4.31 (0.51)	4.71 (0.28)	***p* < 0.001**	***p* < 0.001**	***p* < 0.001**
Perceived collaborative potential	2.85 (0.52)	4.41 (0.32)	3.98 (0.59)	4.79 (0.18)	***p* < 0.001**	***p* < 0.001**	***p* < 0.001**
Conceptual knowledge gain	5.15 (9.56)	16.92 (9.28)	3.00 (9.41)	13.48 (10.81)	*p* = 0.137	***p* < 0.001**	*p* = 0.723

The table displays means with standard deviations in parentheses. im. = immersion, int. = interactivity. *P* values are shown for main effects and interaction effects, with significant effects in bold.

Two-way ANOVAs revealed a significant Immersion by Interactivity interaction for several CAMMT process variables. For control and active learning, the interaction was significant, *F*(1, 113) = 30.61, *p* < 0.001, ηp^2^ = 0.21, and the interactivity effect was larger under low immersion (d = 2.65) than high immersion (d = 0.90), suggesting diminishing returns when both affordances were simultaneously high. Both main effects were significant (immersion: *F*(1, 113) = 124.23, *p* < 0.001, ηp^2^ = 0.52; interactivity: *F*(1, 113) = 99.85, *p* < 0.001, ηp^2^ = 0.47), confirming H3a and H3b. A similar pattern emerged for reflective thinking, with a significant interaction, *F*(1, 113) = 24.86, *p* < 0.001, ηp^2^ = 0.18, and a larger interactivity effect under low immersion (d = 2.19) than high immersion (d = 0.93). Both main effects were significant (immersion: *F*(1, 113) = 85.77, *p* < 0.001, ηp^2^ = 0.43; interactivity: *F*(1, 113) = 79.69, *p* < 0.001, ηp^2^ = 0.41), confirming H4a and H4b. Perceptual-motor skills also showed a significant interaction, *F*(1, 113) = 23.15, *p* < 0.001, ηp^2^ = 0.17, with a larger interactivity effect under low immersion (d = 2.25) than high immersion (d = 1.06). Both main effects were significant (immersion: *F*(1, 113) = 102.98, *p* < 0.001, ηp^2^ = 0.48; interactivity: *F*(1, 113) = 88.01, *p* < 0.001, ηp^2^ = 0.44), confirming H5a and H5b.

**Figure 7:**
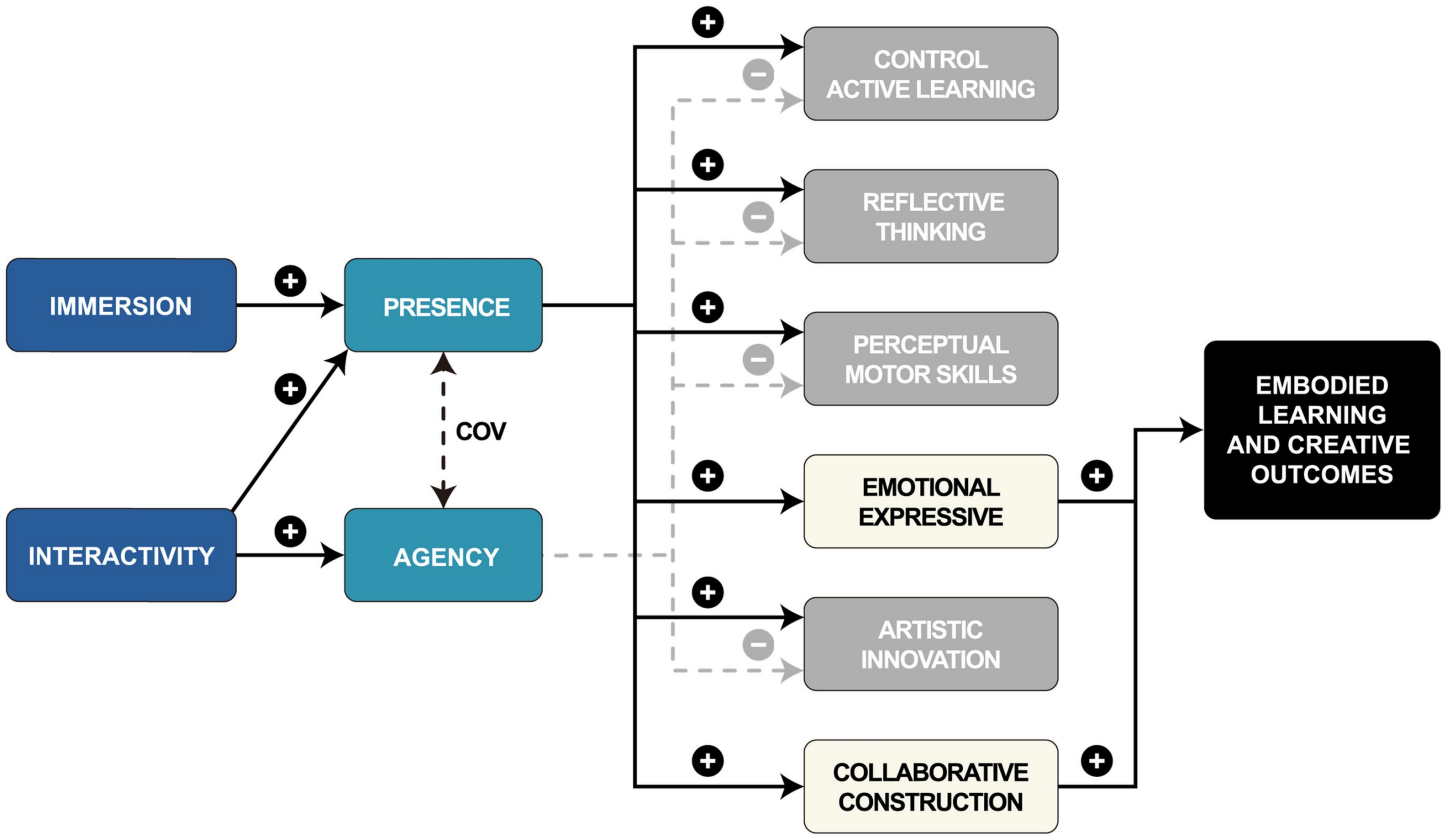
The respecified exploratory CAMMT pathway model.

For emotional expressiveness, there was no significant interaction, *F*(1, 113) = 0.02, *p* = 0.893, but both main effects were significant (immersion: *F*(1, 113) = 29.37, *p* < 0.001, ηp^2^ = 0.21; interactivity: *F*(1, 113) = 102.99, *p* < 0.001, ηp^2^ = 0.48), confirming H6a and H6b. Artistic innovation and perceived collaborative construction potential also showed significant interactions (H7: *F*(1, 113) = 30.66, *p* < 0.001, ηp^2^ = 0.21; H8: *F*(1, 113) = 18.83, *p* < 0.001, ηp^2^ = 0.14), with stronger interactivity effects under low immersion, and significant main effects for both factors, confirming H7a/H7b and H8a/H8b. Finally, for conceptual knowledge gain, immersion was not significant, *F*(1, 113) = 2.24, *p* = 0.137, and the interaction was not significant, *F*(1, 113) = 0.13, *p* = 0.723. However, interactivity had a significant positive effect, *F*(1, 113) = 37.39, *p* < 0.001, ηp^2^ = 0.25. Thus, H9b was confirmed, whereas H9a was not confirmed (Figure 7).

### 4.4 Structural equation modeling (SEM)

Three common goodness-of-fit indicators were used to assess the fit between the hypothesized SEM and the observed data: the Root Mean Square Error of Approximation (RMSEA), the Comparative Fit Index (CFI), and the Tucker-Lewis Index (TLI) (SRMR is also reported as a supplementary index). The hypothesized model’s fit was not acceptable at first (RMSEA = 0.411, CFI = 0.536, TLI = 0.227; SRMR = 0.209). Hence, a theory-consistent respecification was conducted to account for shared variance among conceptually close, same-method self-report constructs (not shown as curved covariances in the graphical illustration of the respecified model). Specifically, the respecified model allowed (a) one covariance between Presence and Agency, and (b) correlated residuals among the six training-process variables (Control Active Learning, Reflective Thinking, Perceptual Motor Skills, Emotional Expressiveness, Artistic Innovation, and Perceived Collaborative Construction Potential). In total, this respecification freed 16 additional covariance parameters: 1 covariance between Presence and Agency and 15 pairwise residual covariances among the six training-process variables. These adjustments are psychometrically and theoretically plausible because the six training-process constructs were measured using the same questionnaire at the same time point and are expected to share method variance and/or omitted common determinants beyond Presence and Agency. The respecified model yielded substantially better CFI than the baseline model (RMSEA = 0.226, CFI = 0.928, TLI = 0.766; SRMR = 0.125), and the improvement relative to the baseline model was significant. Nevertheless, RMSEA, TLI, and SRMR remained outside commonly cited thresholds. Therefore, the SEM results should be interpreted as an exploratory and partial pathway test of CAMMT rather than as a fully validated final model.

#### 4.4.1 Predictions 1 through 3

Presence was positively predicted by immersion (*β* = 0.231, *p* < 0.001) and interactivity (*β* = 0.775, *p* < 0.001). Agency was positively predicted by interactivity (*β* = 0.872, *p* < 0.001). Thus, Predictions 1, 2, and 3 were supported.

#### 4.4.2 Predictions 4 through 15

Across the six training-process variables, Presence was a consistently positive predictor: Control Active Learning (*β* = 0.965, *p* < 0.001), Reflective Thinking (*β* = 0.849, *p* < 0.001), Perceptual Motor Skills (*β* = 0.888, *p* < 0.001), Emotional Expressiveness (*β* = 0.651, *p* < 0.001), Artistic Innovation (*β* = 0.798, *p* < 0.001), and Perceived Collaborative Construction Potential (*β* = 0.820, *p* < 0.001). Thus, Predictions 4, 6, 8, 10, 12, and 14 were supported.

In contrast, Agency did not show the expected uniformly positive effects once Presence was included in the same equations. Agency was not a significant predictor of Emotional Expressiveness (*β* = 0.074, *p* = 0.471) or Perceived Collaborative Construction Potential (*β* = −0.072, *p* = 0.442). Moreover, several Agency paths were statistically significant but in the opposite direction than predicted (i.e., negative unique effects controlling for Presence): Control Active Learning (*β* = −0.390, *p* < 0.001), Reflective Thinking (*β* = −0.291, *p* = 0.009), Perceptual Motor Skills (*β* = −0.296, *p* = 0.006), and Artistic Innovation (*β* = −0.256, *p* = 0.025). Therefore, Predictions 5, 7, 9, 11, 13, and 15 were not supported as stated, and several Agency effects emerged opposite to the expected direction. These negative unique coefficients should be interpreted cautiously because they may reflect shared variance or suppression involving Presence and Agency.

#### 4.4.3 Predictions 16 through 21

Conceptual knowledge gain was positively predicted by Emotional Expressiveness (*β* = 0.419, *p* = 0.004) and Perceived Collaborative Construction Potential (*β* = 0.374, *p* = 0.024). The remaining paths to conceptual knowledge gain were not significant: Control Active Learning (*β* = 0.135, *p* = 0.465), Reflective Thinking (*β* = −0.355, *p* = 0.183), Perceptual Motor Skills (*β* = 0.078, *p* = 0.775), and Artistic Innovation (*β* = −0.341, *p* = 0.124). Thus, Predictions 19 and 21 were supported, whereas Predictions 16, 17, 18, and 20 were not supported. It is noteworthy that Reflective Thinking and Artistic Innovation showed negative (but non-significant) coefficients, which runs counter to the hypothesized positive direction and is considered further in the Discussion.

## 5 Discussion

The aims of the present work were twofold: (1) to investigate the effects of MoCap’s two defining features, interactivity and immersion, on trainees’ learning experience and conceptual knowledge gain, and (2) to provide an initial empirical test and refinement of CAMMT, a theoretical model describing how immersive MoCap training technologies may support learning. Several substantial findings emerged for both objectives. We first discuss the empirical effects of interactivity and immersion on key psychological affordances and training-process variables and then relate these patterns to the SEM results to evaluate and refine CAMMT.

### 5.1 Interactivity and immersion as drivers of presence and agency

A key convergence with prior immersive-learning research is that immersion increased presence, and interactivity increased presence even more strongly. Presence showed significant main effects of immersion, *F*(1, 113) = 16.67, *p* < 0.001, ηp^2^ = 0.13, and interactivity, *F*(1, 113) = 187.78, *p* < 0.001, ηp^2^ = 0.62, with no Immersion by Interactivity interaction. This aligns with presence research linking immersion to presence while emphasizing that presence is shaped by user–system coupling rather than display immersion alone (Witmer and Singer, 1998; Cummings and Bailenson, 2016). It also mirrors process-oriented immersive learning frameworks (Makransky and Petersen, 2021) that treat immersion and control-related interactivity as upstream drivers of presence and agency rather than assuming direct media effects on performance. For agency, the primacy of interactivity was even clearer. Interactivity had a large main effect on agency, *F*(1, 113) = 517.91, *p* < 0.001, ηp^2^ = 0.82, providing strong support for accounts of agency as the experience of initiating and controlling actions and their consequences (Moore and Fletcher, 2012). Agency also showed an important interaction, *F*(1, 113) = 45.91, *p* < 0.001, ηp^2^ = 0.29, indicating that immersion does not automatically enhance agency; immersion amplified agency when action-based control was available.

One striking and researcher-relevant result was that immersion was not uniformly beneficial for agency. Under low interactivity, immersion was associated with lower agency (VR-video < Video), whereas under high interactivity, immersion increased agency (VR-MoCap > MoCap). A plausible explanation is that agency depends heavily on control cues and sensorimotor contingency rather than immersive presentation alone. In an immersive but passive experience, learners may feel situated in the environment but unable to influence it, producing a mismatch that depresses agency. This is consistent with immersive-learning work suggesting that immersive environments can impose attentional and cognitive demands that do not necessarily support learning-relevant control (Parong and Mayer, 2018). Accordingly, immersion should not be treated as a standalone upgrade; its benefits for agency appear conditional on action-enabled interactivity, matching CAMMT’s emphasis on MoCap as embodied, performative learning rather than passive viewing (Jiang et al., 2026).

### 5.2 Training-process variables and learning outcomes

Across the CAMMT training-process variables, the factorial results showed pervasive benefits of immersion and interactivity, with repeated interactions indicating diminishing returns when both affordances were simultaneously high. This pattern is consistent with process-based accounts arguing that media features influence learning indirectly through intermediate states and behaviors, and that increases in technology do not always yield linear gains once key affordances are strong (Moreno and Mayer, 2007; Makransky and Petersen, 2021). These findings also align with MoCap-in-education work framing MoCap as an embodied learning platform, and with the performance-oriented view of animation training in which competence is developed through embodied practice and iterative refinement (Thomas and Johnston, 1995; Wilson, 2002; Barsalou, 2020). At the same time, the present outcome measure captured conceptual knowledge rather than complete animation competence or final artifact quality. A major point of potential inconsistency with “VR improves learning” narratives is that immersion did not significantly increase conceptual knowledge gain, whereas interactivity did. Specifically, knowledge gain showed no immersion effect and no interaction, but a significant main effect of interactivity, *F*(1, 113) = 37.39, *p* < 0.001, ηp^2^ = 0.25. This fits with evidence that VR often increases presence and engagement, while learning effects are heterogeneous and design-dependent, especially for brief interventions and declarative outcomes (Parong and Mayer, 2018; Radianti et al., 2020). In contrast, the interactivity effect is consistent with cognitive-affective (Moreno and Mayer, 2007) and motor learning (Shadmehr et al., 2010) perspectives: active generation, immediate feedback, and iterative adjustment support learning through generative processing and prediction-error correction.

SEM provided a process-level test of selected CAMMT pathways. Consistent with CAMMT’s core premise, SEM showed that immersion and interactivity predicted presence, and interactivity predicted agency. The explained variance was strong for presence (approximately R^2^ = 0.64) and agency (approximately R^2^ = 0.76), indicating that the manipulations mapped cleanly onto these affordances. However, because the respecified model still showed weak RMSEA, TLI, and SRMR values, these findings should be treated as partial support rather than full model validation.

Presence emerged as a consistently strong predictor of all six downstream training-process constructs, supporting CAMMT’s emphasis on performative presence as a catalyst for sustained engagement and refinement. A key unexpected SEM result was that several agency paths were significant but negative once presence was included. This does not necessarily imply that agency is harmful. One plausible interpretation is shared variance and suppression: presence and agency are conceptually adjacent and empirically related, so regression-based models can yield negative unique coefficients when the shared component is partialed out, consistent with the need to include a Presence with Agency covariance to improve fit. A complementary interpretation is that authorship demands may increase self-evaluation standards, performance pressure, and error awareness in a short MoCap lesson, potentially lowering some self-reports in the short term. A third possibility is model misspecification: presence and agency may operate as a coupled embodiment system, as sequential affordances, or through moderated rather than parallel effects. Together, these patterns suggest that agency may operate as part of a coupled system with presence, as a conditional amplifier, or as a moderator rather than as an independent downstream driver in all contexts. Future research should report bivariate correlations, use latent-variable measurement models, and test alternative CAMMT specifications to distinguish these explanations.

SEM also indicated that only emotional expressiveness and perceived collaborative construction potential significantly predicted conceptual knowledge gain. This pattern is theoretically meaningful rather than merely anomalous. In a short conceptual lesson, knowledge gain may depend less on broad self-reported activity or motor-skill confidence and more on whether learners emotionally organize movement meaning and perceive the training method as socially discussable, shareable, and pedagogically meaningful. Emotional expressiveness may help learners connect abstract motion principles to character intention and affect, while perceived collaborative potential may reflect whether learners view the method as a resource for explanation, critique, and co-construction. This does not imply that reflective thinking, perceptual-motor skills, or artistic innovation are unimportant for animation training; rather, these constructs may predict practical artifact quality, expert-rated performance, or longer-term transfer better than immediate conceptual knowledge gains. This interpretation is coherent with cognitive-affective learning theory, which holds that affective engagement can support attention and meaning making when pedagogically guided, and with collaborative learning accounts emphasizing shared inquiry and co-construction (Fischer et al., 2002; Moreno and Mayer, 2007).

### 5.3 Theoretical implications

This study provides an initial empirical test of CAMMT, a recently published theoretical model concerning the process of training with immersive and interactive MoCap technologies that builds on a review of relevant theories and prior immersive learning research (Jiang et al., 2026). The findings favor a feature-based view of immersive MoCap training in which immersion and action-contingent interactivity are distinct affordances with non-additive effects, offering guidance for how future training that integrates these technologies might be designed. Presence increased robustly with immersion and especially with interactivity, highlighting the centrality of action-perception coupling alongside display immersion (Moreno and Mayer, 2007; Cummings and Bailenson, 2016; Makransky and Petersen, 2021). Agency was contingent: high immersion without meaningful control can produce an immersion-control mismatch that reduces perceived authorship, consistent with cue-integration models emphasizing action-outcome contingency and predictive alignment (Moore and Fletcher, 2012; Parong and Mayer, 2018). Moreover, the repeated diminishing-returns interactions suggest nonlinearities and partial redundancy among immersive affordances in performance-oriented learning environments.

Within CAMMT, the substantial association between presence and agency, together with the dominant downstream influence of presence, supports the view that these affordances may operate as an integrated embodiment system. Accordingly, agency’s heterogeneous unique coefficients in the SEM are more plausibly attributed to shared variance/suppression dynamics, model misspecification, and/or heightened authorship demands that amplify error awareness during training than to a simple harmful effect of agency. Finally, emotional expressiveness and perceived collaborative construction potential most strongly predicted conceptual knowledge gain, implying that conceptual learning in creative-performance domains may be closely tied to expressive externalization and social meaning-making. These results motivate CAMMT refinements that emphasize conditional pathways and conversion mechanisms via iterative expression, critique, and collaborative potential, while acknowledging that the present SEM offers partial rather than definitive support.

### 5.4 Limitations and suggestions for future research

An important limitation of the present study concerns the specific virtual learning environment and instructional goal that were selected. As noted above, the lesson was designed primarily to teach declarative knowledge related to character animation, and learning outcomes were operationalized as participants’ improvement on a character-animation knowledge test. While this design enabled us to examine how immersion and interactivity-related variables influence the learning process of understanding motion principles, it also constrains the generalizability of the findings. Animation training, and computer animation training in particular, involves not only conceptual understanding but also procedural performance, motion design competence, creative decision-making, critique, and final artifact quality. Therefore, the findings should not be interpreted as evidence that MoCap instruction fully improves animation skill acquisition. Future research should retain knowledge-based measures while also incorporating outcomes that better reflect the nature of animation training, such as motion design quality, creative diversity, expressive performance, transfer tasks, and expert-rated artifacts. In addition, if the instructional goal is to teach procedural skills (e.g., how to operate a device or software), the pathways to learning may differ.

A related methodological limitation concerns the interpretation of the interactivity manipulation. The high-interactivity conditions required learners to enact and control character motion through MoCap, whereas the low-interactivity conditions involved observation of the same content without action control. Consequently, the manipulation necessarily combined technological interactivity with embodied practice. The study therefore should not be interpreted as isolating interactivity from all active-learning components. Rather, the findings concern action-contingent MoCap interactivity as implemented through embodied performance and real-time feedback.

Another limitation concerns classroom relevance. The video and VR-video conditions served as controlled experimental baselines and should not be read as representations of ideal animation pedagogy. In actual animation courses, conceptual instruction is usually integrated with practical production exercises, critique, iterative revision, and assessment of artifacts. Future studies should therefore include mixed-method or triangulated designs, such as post-lesson animation tasks, expert ratings of motion quality, student reflection logs, or interviews with learners and instructors.

A further limitation concerns the interpretation of the SEM results. Although SEM is a powerful method for testing theory-consistent relationships among variables, a common criticism is that it can be used to imply causality on the basis of association alone; in practice, SEM does not prove causal hypotheses but can only increase their plausibility when the model is theoretically well justified (Bollen and Pearl, 2013). Moreover, the structural model being tested necessarily reflects the researcher’s causal assumptions, which are grounded in prior research, scientific knowledge, and logical argumentation. In the present study, these assumptions were derived from a research-based theoretical framework, yet the SEM relied on composite scores rather than full latent measurement models, which limits tests of discriminant validity, particularly for closely related constructs such as presence and agency. The respecified SEM also required 16 additional covariance parameters and still retained weak RMSEA, TLI, and SRMR values; therefore, the results should be interpreted as partial and exploratory. Because CAMMT was proposed by the same lead author team, the present study also represents an initial author-led empirical test of the model. Future independent replications, bivariate correlation reporting, and latent-variable SEM with stronger measurement modeling are needed to further strengthen confidence in the framework’s generalizability.

A further limitation is that collaborative construction was measured as perceived collaborative potential rather than actual peer interaction. Participants were explicitly instructed not to interact during the experiment; therefore, the construct should not be interpreted as evidence that collaboration occurred during the session. Future studies should test CAMMT in genuinely collaborative MoCap settings and should examine whether perceived collaborative potential differs from observed collaborative behavior.

Finally, total session duration differed across conditions because MoCap and VR-MoCap required equipment setup and calibration. Although core instructional content was held constant, the additional setup time, novelty of equipment, and preparation process may have influenced learners’ experiences. Future experiments should log instructional exposure, setup/calibration time, and active engagement time separately, or should equalize total exposure across conditions.

It is also worth noting the limitation concerning contextual and individual factors that can substantially shape learners’ experiences in MoCap-based training but are not explicitly represented in CAMMT. These include system usability, social influences, and learner characteristics such as age, personality traits, spatial ability, and prior experience with immersive systems. Accounting for such moderators is important for explaining variability in MoCap-based creative performance and innovation outcomes. In addition, although CAMMT does not currently include cognitive load, this construct is widely regarded as central in research on how novel technologies affect learning and can even help predict learning outcomes (Albus et al., 2021; Petersen et al., 2022; Schrader et al., 2024). Future studies should therefore incorporate cognitive-load measures and test whether they mediate or moderate training effects.

Individual differences may further moderate MoCap training effects. For example, younger learners may show greater openness to immersive technologies (Suh and Prophet, 2018), and spatial ability may influence how effectively learners manipulate and interpret motion information in 3D environments (Li et al., 2020). Conversely, physical fatigue, motion discomfort, or body-image anxiety may reduce engagement by constraining embodied participation. These considerations highlight the need for inclusive and ergonomic design that accommodates diverse physical and psychological profiles to support equitable access to creative education. Finally, the sample in this study had an unequal gender distribution, approximately 60% female, which is common among art students; while there is no clear reason to assume that this alone biased the results, the reliance on animation students from a single university may limit external validity because they may constitute a special group of learners.

## 6 Conclusion

Overall, the study provides convergent but bounded evidence that action-contingent interactivity is the primary driver of MoCap training benefits, improving presence, agency, and conceptual knowledge gain, whereas immersion robustly improves presence and training-process experiences but does not guarantee improved conceptual knowledge gain in short lessons. SEM provides partial support for CAMMT by confirming the upstream affordance pathways and establishing presence as the dominant predictor of downstream training-process variables, while also identifying pathways that require refinement. The conditional immersion effect on agency and the unexpected agency patterns in SEM indicate that, in performance-driven MoCap learning, agency appears tightly intertwined with presence and is most beneficial when immersion is paired with action-enabled control. Crucially, within one experiment with four conditions, this study delineates how MoCap’s technological affordances and the psychological dynamics they evoke may jointly shape conceptual animation learning. These findings suggest that motion capture can be more than an entertainment-oriented add-on when it is pedagogically integrated, while also showing that its benefits depend on the alignment between technology, practice, outcome measures, and classroom assessment. The study therefore provides a springboard for future research on the use of MoCap in animation learning and skills training.

## Data Availability

The raw data supporting the conclusions of this article will be made available by the authors, without undue reservation.
