# Supplementary material for: A study of how immersion and interactivity in MoCap mediate psychological affordances and drive conceptual learning in computer animation courses
Source: Front Psychol. 2026 Jun 9;17:1813127. doi: 10.3389/fpsyg.2026.1813127 (PMC13288770; doi:10.3389/fpsyg.2026.1813127)
Supplement: Supplementary file 1 [file Supplementary_file_1.docx]

**Appendix A**

| Construct | Item | Questions | source |
| --- | --- | --- | --- |
| Presence | p_1 | During training, I feel that my movements are realistically mapped onto the virtual character. |  |
|  | p_2 | During training, I feel a sense of immersion, as if I were truly performing in the virtual world. | Makransky et al., 2017 |
|  | p_3 | My real movements can be naturally integrated into the animation production process. |  |
|  | p_4 | During training, I do not feel a connection between myself and the virtual character. |  |
| Agency | A_1 | During training, I have a strong sense of control over the animated character’s movements. | Polito et al., 2013 |
|  | A_2 | The training can accurately reflect my physical movements. |  |
|  | A_3 | The training makes me feel that I have full autonomy to design the animation. |  |
|  | A_4 | During training, I feel that my control over the animated character is weak. |  |
| Control Active Learning | CAL_1 | The training prompts me to try different animation techniques. |  |
|  | CAL_2 | Based on the real-time feedback provided by this training approach, I proactively adjust my movements to improve the animation outcome. | Lee et al., 2010 |
|  | CAL_3 | During training, I often become fully absorbed in creating animation. |  |
|  | CAL_4 | By actively engaging in this training, I feel motivated to improve my animation skills. |  |
|  | CAL_5 | During training, I rarely take the initiative to adjust my movements. |  |
| Reflective Thinking | RT_1 | During training, I often reflect on how my movements affect the animation results. | Maor & Fraser, 2005 |
|  | RT_2 | The training enables me to systematically analyze and improve my animation techniques. |  |
|  | RT_3 | Viewing the movements recorded helps me critically evaluate my animation performance. |  |
|  | RT_4 | The training encourages me to think deeply about the character’s movement design. |  |
|  | RT_5 | During training, I rarely review and reflect on my animation performance process. |  |
| Perceptual Motor Skills | PMS_1 | During training, it improves my ability to reproduce realistic character motion. |  |
|  | PMS_2 | The training helps me understand the biomechanics principles of human movement. |  |
|  | PMS_3 | The training enhances my spatial perception in animation production. | Rovai et al., 2009 |
|  | PMS_4 | During training, it improves my bodily coordination. |  |
|  | PMS_5 | During training, it does not help me improve my understanding of character movement. |  |
| Emotional Expressive | EE_1 | Performing animation within the training system makes it easier for me to form an emotional connection with the character. | Kring et al., 1994 |
|  | EE_2 | The training enables me to explore and refine the character’s emotional expression more deeply. |  |
|  | EE_3 | Training in this system allows me to invest more emotion in the animation creation process. |  |
|  | EE_4 | The training enables me to give the character more personality and emotion. |  |
|  | EE_5 | In the training, I feel that the character’s emotional expression is rather stiff. |  |
| Artistic Innovation | AI_1 | The training opens new creative possibilities for character animation. | Cherry & Latulipe, 2014 |
|  | AI_2 | This training allows me to experiment with different animation styles and freely adjust motion data. |  |
|  | AI_3 | This training makes it more likely for me to develop a unique animation style. |  |
|  | AI_4 | This training encourages me to break through traditional constraints and innovate in animation performance. |  |
|  | AI_5 | This training limits my ways of creating animation, making them less diverse. |  |
| Collaborative Construction | CC_1 | Using this training approach in a team setting can enhance my learning experience. |  |
|  | CC_2 | Sharing this training approach with others helps me better understand character motion. | So & Brush, 2008 |
|  | CC_3 | Collaborating in such a training environment facilitates knowledge exchange among animators. |  |
|  | CC_4 | This training supports group learning, enabling members to adjust and discuss animation creation through real-time interaction. |  |
|  | CC_5 | During this training approach, I rarely collaborate or communicate with others during animation training. |  |

**Knowledge Test**

| No. | Question | Options |
| --- | --- | --- |
| 1 | What does anticipation help achieve in animation? | A) Preparing for an upcoming action B) Accelerating the action C) Increasing the speed of the action D) Reducing the number of frames |
| 2 | What does the staging principle in animation mainly ensure? | A) The action is easy to understand B) The character moves quickly C) The background is detailed D) The visual focus is maintained |
| 3 | What do “slow-in” and “slow-out” describe in animation? | A) Emotional changes B) Changes in speed during movement C) Scene transitions D) The naturalness of the movement |
| 4 | Why is arc motion important in animation? | A) It ensures movements look realistic B) It makes animation faster to produce C) It conveys natural movement D) It is easier to render |
| 5 | What role do secondary actions play in animation? | A) Distracting attention from the main action B) Supporting and enhancing the main action C) Adding depth to the scene D) Speeding up the main action |
| 6 | What does “straight-ahead” describe in animation? | A) Animating frame by frame from start to finish B) Using only key poses C) Ignoring background details D) Allowing improvisation |
| 7 | In animation, what aspect of movement does “follow-through” refer to? | A) Movement that continues after the main action B) Initial acceleration C) Enhancing the realism of the action D) Only the main character’s movement |
| 8 | In animation, what does timing and rhythm affect? | A) The number of frames in a movement B) Emotional pacing C) The color scheme D) Plot development |
| 9 | What is the purpose of exaggeration in animation? | A) Enhancing dramatic or comedic effects B) Reducing visual clarity C) Achieving photo-realism D) Making movements more expressive |
| 10 | What ensures an animated character’s appeal? | A) Visual and emotional attractiveness B) Consistency in character design C) Consistent movement patterns D) Distinct personality traits |
